# An assessment of a ‘training-of-trainers programme for clinic committees’ in a South African district: a qualitative exploratory study

**DOI:** 10.1186/s12913-020-05921-z

**Published:** 2020-11-30

**Authors:** Natasha Esau, René English, Maylene Shung-King

**Affiliations:** 1 Health Systems Trust, 1 Maryvale Road, Westville, PO box 784, Durban, 3630 South Africa; 2 Health Policy and Systems Division, School of Public Health and Family Medicine, University of Cape Town, Falmouth Road, Observatory, Cape Town, 7925 South Africa; 3 Division of Health Systems and Public Health, Department of Global Health, Stellenbosch University, Private 1243 Bag X1, Matieland, Stellenbosch, 7602 South Africa

**Keywords:** Governance structures, Clinic committees, Training, Trainer-of-facilitator, South Africa

## Abstract

**Background:**

In South Africa (SA), clinics and community health centres are the predominant primary level health care facilities in the public health sector. As part of legislated health governance requirements, clinic committees (referring to those for clinics and community health centres) were established to provide management oversight and bring to bear the perspectives and participation of communities at Primary Health Care (PHC) facilities. Clinic committees need training in order to better understand their roles. Facilitators in a district of SA were trained through a designated programme, called the ‘PHC Facility Governance Structures Trainer-of-Facilitator (ToF) Learning Programme‘, in preparation for the training of clinic committees. This paper explores how the programme had evolved and was experienced by the trained facilitators, in a district in SA.

**Methods:**

We employed a retrospective qualitative case study design, guided by the Illuminative Evaluation Framework, with the training programme in the selected district as the case. The study assessed whether the intended aims of the training programme were clearly conveyed by the trainers, and how participants understood and subsequently conveyed the training programme intentions to the clinic committees. Key informant interviews and focus group discussions were conducted with trainers and managers, complemented by a review of relevant policy and legislative documents, and published literature.

Study participants were purposively selected based on their involvement in the development, facilitation or training of the programme. Thirteen individuals participated in the study, and 23 (national, provincial and partner) documents were reviewed.

**Results:**

Despite the different perceptions and understandings of the ToF Learning Programme, its overall aims were achieved. Trainers’ capacity was strengthened and clinic committees were trained accordingly. The training programme holds promise for possible national scale-up. The high quality of the interactive posters can be considered equally valuable as a training tool as the training manuals.

**Conclusions:**

Trainers’ capacity was strengthened and clinic committees were trained accordingly, despite deviations in implementation of the original training approach and plan.

## Background

Leadership and governance are essential building blocks of a good health system [1] and an essential dimension of health system governance is the participation and contribution of communities. The Alma Ata Declaration, following the international conference on Primary Health Care (PHC) in 1978, foregrounded the importance of community participation in health, and health authorities’ responsibility to capacitate community members to this end [2].

Formal structures, such as clinic committees, have been put in place globally to provide a vehicle for community participation and to assist facilities to execute their health service mandates [1, 3, 4]. However, clinic committees require the necessary capacity to fulfil their governance role effectively. Good, quality and appropriate training is one mechanism through which the capacity of clinic committees can be enhanced [5].

Strides towards capacitating clinic committees, implementing community monitoring approaches and prioritising the establishment and capacitation of communities participating in PHC governance activities have been made by organisations and bodies such as the Community Practitioners on Accountability and Social Action in Health (COPASAH), and Regional Network for Equity in Health in East and Southern Africa (EQUINET). Despite an increase in community participation in health, active engagement in decision-making and planning and execution processes are still hampered by challenges with capacity [6]. For example, in Kenya, health facility management committees were expected to engage in financial management tasks, but fewer than 18% of committees, and only certain individuals, reportedly received facility and/or financial management training [7].

A key barrier to ongoing training and capacity building relates to training initiatives often being financially unsustainable [8]. Other factors, according to Rifkin (2009), include: the pre-eminence of the bio-medical approach that reduces community participation to an intervention; inadequately understanding the perceptions of the community; and not employing a framework for capacity development that recognises the positive contribution of community participation [9].

A systematic review by McCoy et al. found that in Peru, Zimbabwe, Kenya and Uganda, clinic committees can positively contribute towards health outcomes [10]. The review also showed that facilities with committees had better service utilisation, user satisfaction and access to health care for the poor. Committees also improved health workers’ performance, despite not always functioning in a governance capacity and the negative perceptions of officials and health professionals towards them [10]. McCoy et al. highlights the usefulness of introducing a framework that clarifies the roles and responsibilities of clinic committees, which includes governance, co-management, resource generator, community outreach, advocacy, intelligence, and social leveller [10].

Further recommendations are that train-the-trainer capacity building approaches, particularly those directed at clinic committees, should take into account issues such as resources, a transferable curriculum, skilled facilitators, clear purpose and outcomes and efforts to promote sustainability [11–15].

### South African context

A central goal of health system reform in South Africa (SA) over the past 25 years has been the establishment of a decentralized district health system that would meet the needs of all citizens [16]. The *White Paper for the transformation of the health system in South Africa* released in 1997 advocated for community participation in health governance, thus requiring the establishment of governance structures that would enable this [16]. In SA the concept of health governance refers to ‘clinic committees, community health-centre committees, hospital boards and district health councils’, these being the key structures that allow for community participation in governing health facilities [17].

Provinces, through their provincial legislation and as required in the National Health Act of 2003 were tasked with the establishment of committees for clinics and community health centres, this required a clarification of their roles and functions [18]. In the study district, clinic committee members are nominated by the community and elected by the Member of Executive Council for Health (MEC). The positions are advertised and eligible community representatives are invited to attend nomination meetings [19]. The purpose of the clinic committee is to help the PHC facility meet its responsibilities, to make sure that PHC facility management meet its obligations and to see to it that the PHC facility is responsive to community needs [19]. Members serve a three year term, which is renewable for a second term and the committee is expected to meet on a quarterly basis [19]. Participation in clinic decision-making is mainly done through clinic committee membership.

Despite some policy guidelines on the structure and function of committees, a number of factors impede the effective functioning of clinic committees [20]. These include the composition of committees, poor adherence to existing policy guidelines, members not always operating according to the stipulated functions, member election processes not always being transparent, no clear linkages to the broader governance structures or other government sectors, and inadequate financial and technical input and lack of training [20].

In SA, community members appointed to health governance structures are meant to function within a governing role (i.e. overseeing facility management processes) alongside facility managers. Yet, a recent study conducted in a South African province, that included interviews with members of clinic committees, showed that clinic committees generally do not perform governance functions, but menial tasks [21]. Many clinic committee members do not have high school certificates and struggle with literacy and numeracy skills, which makes reading, writing and communication difficult. Yet the latter are required for their day-to-day functioning as committee members.

An assessment on the status of clinic committees at PHC facilities in SA revealed that training of clinic committees remained neglected and often did not meet the requirements for capacitation [20]. The assessment advocated for ongoing, sustainable, training programmes and a more collaborative partnership between the health system and governance structures that involves trust and resources [20, 22]. A study conducted in the Eastern Cape province argued that clinic committees should be platforms for capacity building and personal development, with a systematic plan and direction to increase sustainability and effectiveness of both clinic committees and the necessary training initiatives [22]. There is little evidence that the health system provides the resources for capacitating clinic committees and this ultimately interferes with the delivery of effective health governance and building of the health system [17].

### The case

South Africa’s nine provinces made progress towards meeting the requirements for community participation in health in different ways. Some provinces have formally trained their clinic committees through external contractors. One of these external agencies, a well-established Non-Governmental Organisation (NGO), was contracted by one of the nine provinces in SA to do such training in one of its districts (the district of focus for this study).

In order for clinic committees in this district to understand their role better and to be adequately capacitated, they were earmarked to undergo training. The district Department of Health (DDoH) identified potential district level trainers; district and sub-district managers and health facility supervisors, who in turn would train the clinic committee members. Towards the end of 2015, the DDoH requested of the NGO to train its selected trainers. Due to the district’s time and budgetary constraints, a ‘trainer-of-trainer’ approach was deemed best in serving their training needs. Variants in the name of the training exist, thus for the purpose of this study we have named the training ‘Primary Health Care (PHC) Facility Governance Structures Trainer-of-Facilitator (ToF) Learning Programme’ or ‘ToF Learning Programme’.

The training was conducted in 2016 with two facilitators and 53 participants. The facilitators were employed by the NGO. The training formed part of their scope of practice, thus they did not receive extra financial compensation. The newly trained facilitators were employees of the Provincial Department of Health and the training was a means of capacitating them to perform their duties. No extra financial compensation was afforded to them. This approach allows for in-house capacity development that allows for future training to be done using existing human resources, with no additional costs incurred. In 2014, the NGO in consultation with the National Department of Health (NDoH) developed the ToF Learning Programme structure and training materials, which are outlined in Table 1. The programme has four overall learning outcomes in three modules and each module has a specific set of training material serving a specific purpose (see Table 1). The overall aim of the ToF Learning Programme is to strengthen the facilitator’s capacity to deliver the PHC facility governance structure capacity strengthening learning programme, to the clinic committees [23].

**Table 1:** ToF Learning Programme Training Material

	Purpose and participants	Material	Purpose of material
**Module 1**	Training of facilitators	Facilitation guide for capacity strengthening of health governance structures	•Provides background knowledge of health governance, facilitation and adult education •Introduces the ToF Learning Programme to facilitators •Provides guidance on how to use the guide and how to facilitate the ToF Learning Programme
**Module 2**	Training of clinic committees	Learning resources for PHC facility governance structure members	•To be used together with the facilitators guide to train governance structure members
**Module 3**	Follow-up sessions and on-going mentoring of clinic committees	1. A set of 16 Posters 2. Pocket Handbook	•Used for reflection of the training, highlighting areas needing clarification •To be displayed in health facilities for easy access and reference to governance structure members and health facility staff •An easy reference guide of roles and responsibilities for clinic committees, health managers and facility staff •To be used during follow-up sessions
Four overall learning outcomes 1. Understand the legislative and administrative framework and organisational structure of the NHS 2. Participate in and contribute to the planning, M&E, and quality assurance of health establishments 3. Understand health paradigm 4. Use and apply relevant information, knowledge, skills and values to fulfil the governance role in the health system			

Source: Health Systems Trust

The ToF Learning Programme is based on the principles of Adult Education Theory (AET). AET is transformative and autonomous as it facilitates critical thinking and the assessment of knowledge in order to derive one’s own conclusions regarding a particular area of interest [24]. It is the art of facilitating learning to adult learners, through specifically crafted activities [25]. The ToF Learning Programme was participatory in nature which comprised of various discussions, role-play scenarios and feedback sessions.

The programme was first piloted in another district in 2015 by the NGO team. During this pilot, the training programme was implemented as initially intended. The role of the NGO training facilitators was to train the district’s master trainers or trainees (Module 1) (see Table 1). They were also to oversee the training of the clinic committees (Module 2), follow-up sessions and the ongoing mentoring (Module 3) as facilitated by the trainees. A short evaluation at the end of this pilot revealed areas in the programme that needed revision. The NGO team had subsequently incorporated these changes.

However, in the study district due to budgetary and capacity constraints, the NGO facilitators were only contracted to facilitate the training of facilitators (Module 1). Upon completion of their training, the facilitators implemented Module 2 in their own sub-districts and Module 3 at a later stage without the oversight of the NGO facilitators.

Given the change in the original training plan, a request from the NGO was made for an assessment of whether the model of leaving newly trained trainers behind works, as opposed to having the NGO facilitators facilitate the process through modules one to three. This study is in response to this request and assessed the ToF Learning Programme in the study district and explored: the context in which the training occurred whether the training was done according to the intentions of the ToF Learning Programme; and whether the districts selected trainers understood and were able to apply the training to the clinic committees. The study explored the training of the trainers only and not the application of the training with the clinic committees. The study was conducted in 2017 and the ultimate goal was to illuminate whether the training had the potential for wider application in SA.

## Methods

### Study design

This was a retrospective qualitative single case study, in which the case was the implementation experience of the ToF Learning Programme in one district. We conducted key informant interviews (KIIs), focus group discussions (FGDs) and a document review. A KII and FGD questionnaire was developed for this study (see additional file 1). The perspectives of the trainers on how they experienced the training, and how they perceived the clinic committees to have experienced the training, were explored. The outcomes of the training for the clinic committees and the perspectives of the clinic committee members were not considered at this stage and may be explored as a possible follow-up study at a later stage. The study was guided by the Illuminative Evaluation Framework (IEF).

### Study setting

The study was conducted in one district in one of South Africa’s nine provinces. The district consists of six sub-districts and has a catchment population of 1,357,744 (Midyear estimates, 2013) [26] and 115 health facilities (fixed clinics, community health centres, mobile clinics, hospitals) across its sub-districts. In the province, a total of 188 clinic committees have been established and the study district has a total of 90 established clinic committees consisting of at least ten members each.

### Conceptual framework

The Illuminative Evaluation Framework (IEF), is shown in Table 2 and discussed below.

**Table 2:** Illuminative Evaluation Framework

IEF overview	
Enables researches to →	1. Explore the educational process 2. Explore programme outcomes 3. Explore its consequences
The aims of illuminative evaluation are to study an innovatory programme →	1. How it operates 2. How it is influences by the various situations in which it is applied 3. What those directly concerned think are its advantages and how students’ intellectual tasks and academic experiences are most affected
It seeks to document and discover what it is like to be →	1. Participating in the scheme, whether as student or teacher 2. It looks for the most significant features and critical processes of the innovation (in this case, the ToF Learning Programme)

Source: Taken from Smith, Masterson & Lask, 1995; 246

Other conceptual frameworks considered were the Kirkpatrick and Kirkpatrick’s ‘Evaluating Training Programmes’ [27], Holton’s ‘Human Resource Development (HRD) Evaluation Research and Measurement Model’ [28] and the Buse et al’s ‘Health Policy Analysis’ [29]. These were, however, not suitable as they focussed primarily on the learning experiences of the participants in the training, with not enough emphasis on the context, content, and the process undertaken, or the power dynamics of stakeholders.

The rationale and design for the IEF was introduced by editors Parlett and Dearden in 1977 and looks at a learning programme’s educational processes or the instruction system (course material), programme outcomes or the learning milieu (through qualitative collection of data) and the consequences of the programme [30, 31]. The instruction system also refers to the context within which learning takes place, highlighting the roles of the various actors which enhance aspects of the programme outcomes and its consequences. The IEF continues to be used to evaluate various educational training programmes as well as to inform training and implementation policies [32].

The questions we explored included how the adopted training approach worked in the district, and whether the training approach was sufficient or comprehensive enough for the newly trained facilitators to train the clinic committees, without the assistance of external trainers. The IEF enabled the assessment of the ToF Learning Programme by checking whether the aims, objectives and methodology of the training programme were clearly conveyed by the facilitators, whether this was understood by the participants and whether these participants were able to transfer the training programme, as intended, to the clinic committee members. It also enabled the documentation of participants’ perceptions and experiences of the training.

### Sampling

Participants were purposively selected based on their involvement in the ToF Learning Programme, their availability and consent given to participate in the study. Due to the number of sub-districts and clinic committee members, a total of 53 managers and health facility supervisors participated in the training. Of the 53 trainees who were invited to participate in the study, 11 agreed. Seven of the 11 trainees agreed to participate in two FGDs and the remaining four participated in KIIs. KIIs were held with the two NGO training facilitators, the PHC director and a sub-district manager. The two FGDs consisted of [1] DDoH sub-district managers and [2] health facility supervisors. Even though the study’s methodology did not set out to use snowballing as a sampling technique, during KIIs a further two key informants (NGO and NDoH) were identified as important to the process, based on their involvement in the initial development of the training programme, and included (see Table 3). Participants were informed that their participation in the study was voluntary and confidentiality was assured, hence a safe space was created for participation that allowed participants to engage freely in the discussions. Logistically the FGDs also worked better.

Credibility and rigour of findings were ensured by triangulating the FGDs, KIIs, literature review and document review. Even though the participant number was small the responses converged very quickly. Key informants were available after the interviews and contacted as needed to verify any information deemed unclear. During data analysis, frequent and iterative meetings were held between the lead author and the co-authors to discuss any disagreements until a common agreement was reached.

### Data collection

#### Data collection involved document reviews and interviews

##### Interviews

Interviews involved KIIs and FGDs (see Table 3) of approximately 60–90 min each and these were conducted by the lead author. In person KIIs and FGDs were conducted in the study district during consecutive sessions. One KII was conducted in a different province. Prior to the interviews the participants were each given a study information sheet and consent form, in person or via email. Consent forms sent via email were either signed electronically or signed and scanned and returned via email. After ensuring that the participants read and understood the information sheet and signed the informed consent form, the interviews and discussions were conducted in English and were audio recorded and transcribed verbatim. Confidentiality of all participants (in-person and telephonic) was maintained. No names were mentioned and ranks (positions) were generalised. The FGDs were written up as a collective rather than individual responses. The interview process was semi-structured, with open ended and clarifying questions pertaining to participants’ perceptions and understanding of the training, and whether the learning programme was delivered to the clinic committees. The interview responses were very similar within and between FGDs. Across the two FGDs and the KIIs we felt that saturation was reached and that additional participants were not likely to yield additional or different responses. The FGD participants represented the spectrum of trainers namely sub-district managers and health facility supervisors.

**Table 3:** Sampling

	Department	Description	Number of participants	Interview method	Total participants
**Key informant interviews**	NDoH	•Informal conversation •Based on involvement in the initial development of the training programme	1	Telephonic	6
	DDoH	•PHC Director •Sub-district manager	1 1	Face to face, Face to face	
	NGO	•Director: based on involvement in the initial development of the training programme •Training facilitators	1 2	Face to face, Telephonic	
**Focus group discussions**	**Groups**	**Description**	**Number of participants**	**Interview method**	**Total participants**
	Group 1	Sub-district managers	3	Face to face	7
	Group 2	Health facility supervisors	4	Face to face	

#### Document review

Document reviews are useful means of data triangulation, and is cost-effective [33]. To gain an understanding of the policy and legislative requirements for clinic committees and the subsequent practical application of these in SA, relevant available published and unpublished policies, legislative and appropriate training guidelines were searched for and reviewed. Reports and documents containing the evaluation of the training were not obtainable, even though these were requested from relevant authorities. Inclusion and exclusion criteria are presented in Table 4 below.

**Table 4:** Inclusion and exclusion criteria for the documentary review

Inclusion criteria	Exclusion criteria
1. South African documents: a. policies, laws and training guidelines b. that say something about clinic committees’ role, position and training South African documents or policies 2. Speak to clinic committees specifically: a. Role, position and training 3. Policies and or documents dated between 1996 and 2017	1. Documents: a. not from SA b. speaks to hospital boards or other forms of community participation 2. Policies and or documents outside of the 1996 and 2017 timeframe

Sources of information consisted of publicly available national and provincial government websites, academic institutions and the participating NGO that supports the development of government departments.

A data extraction template was developed using a Microsoft Excel spreadsheet to chart relevant data. Shown in Table 5 below are the search terms used.

**Table 5:** Search terms

Search terms	
“health” AND “policy”, “clinic committees” AND “South Africa”, “health policy”, “South African health policies”, “national” AND “policies” AND “clinic committees”, “clinic committees” AND “training” AND “policy”, “South Africa” AND “clinic committees”, “South Africa” AND “guidelines”, “South Africa” AND “strategic objectives”	

### Data analysis

Due to the small sample size the data were manually analysed, using the methodology presented by Ritchie and Lewis (2003). This iterative form of analysis consisted of three phases: data management, descriptive accounts and explanatory accounts [34].

#### Phase 1: data management

The transcripts were anonymised by assigning unique codes to participants. The transcripts for the FGDs were kept in two separate groups, namely for [1] the sub-district managers, and [2] the health facility supervisors. The KIIs were individually analysed and stored. A coding framework was designed using an Excel spreadsheet (see Table 6 in results section). Deductive themes as informed by the literature review, were the initial set of themes explored in the interviews. In both the transcripts and spreadsheets various parts of the texts were assigned to the various codes and each code assigned a number and were then further analysed until sub-themes and sub-sub-themes emerged. Text was assigned to the subsequent themes in the same manner as for the initial themes. Inductive or new themes also emerged and were systematically explored across all transcripts. The analysis for the inductive theme followed the same procedure as the deductive themes. The lead author conducted the analysis. There were frequent and iterative meetings between the lead author and the co-authors who reviewed and commented on the analysis. Where there were any differences between authors, these were discussed until a common agreement was reached.

**Table 6:** Coding Framework

Themes (initial)	Themes (round 1)	Themes (round 2)	Final themes
Training facilitators	Knowledge and perceptions of clinic committees	Perceptions	Participants’ perceptions of the training
Training material	Perceptions prior to and after the training	Training	Participants’ perceptions and experiences of clinic committees
Training process	Feedback regarding clinic committees	Training material	Capacity building of the trainers
Training of clinic committees	Perceptions of the training	Clinic committees	Empowerment of participants
	Adult education	Empowerment	Ownership of health facilities
	Revision/improvement of the model	Capacitation	Posters as valuable teaching aids for clinic committees
	Naming of training	Ownership	Printing of the training material
	Translation of material	Stipends as motivator	The role of stipends
	Training of facilitators	Challenges	
	Empowerment		
	Capacitation		
	Stipends		

#### Phase 2: descriptive accounts

All themes were collated into one spreadsheet and the content of each theme was categorised and classified into sub-themes [34]. Data were examined for participants’ responses to the themes and sub-themes and how different participants responded to specific themes thus eliciting the variants, similarities, and interesting elements within the different responses. This gave rise to new interpretations and understandings of the themes, which highlighted the interconnectedness between various themes.

#### Phase 3: explanatory accounts

A thematic chart containing summarised versions of the managed and described data was used to explore the identified patterns and associations [34]. After checking the number of times these patterns and associations appeared across the dataset, data were clustered together and explanations were assigned to the patterns. These explanations resulted from the researchers theoretical perspective, inference of an underlying logic, the use of explicit reasoning and using common sense [34].

### Documentary data analysis

Excel spreadsheets were created for deductive themes that arose from within the text. The development of themes was an iterative process and followed the same procedure presented above. The data were then triangulated with the objectives of the study, the qualitative results and the literature review.

## Results

A coding framework highlighting the initial themes and final themes was designed (see Table 6).

### Document review results

The purpose of the document review was mainly to understand the overall policy and legislative environment for clinic committees. A review of South African documents revealed that the establishment of clinic committees is a national requirement. However, the implementation across provinces is not standardised, for example, the issue of stipends for clinic committee members is stipulated by some, whilst others are silent on the issue. Training is another aspect that evolved differently across provinces.

#### The establishment of clinic committees a national requirement

From as early as 1997, community participation in health, through the establishment of governance structures, has been a department of health requirement [16]. This is reflected in all 23 documents reviewed (policies, guidelines, reports and training material). The first province to have legislated clinic committees, in 1999, was the Eastern Cape [35]. The South African National Health Act, of 2003 (NHA), mandated the establishment of clinic committees but offered limited guidance with regards to ‘the how’ and left this up to the provinces [18]. As a result, clinic committees differ across SA in terms of its composition, eligibility criteria for membership, roles and responsibilities, and powers and functions. At least three provincial documents, including the earlier versions of these documents, have not been explicit about the composition of their committees [35–37].

With regards to the composition of committees, some provinces followed the NHA guide: one or more local government councillors, one or more members of the community, and the head of the facility and added a ward councillor and/or municipal councillors [38–41]. Other provinces followed a more intersectoral approach [42]. Some provinces like Mpumalanga, in their guideline, included organized labour, traditional authorities and people representing disabled groups amongst others [35, 43, 44].

A National Colloquium, held in Cape Town in 2014, provided an opportunity for stakeholders working on community participation in health to share research findings and experiences [42]. It concurred that the roles, responsibilities, powers and functions of committees are generally understood to mean oversight, governance, advocacy, social mobilization and representing the needs of the community [42]. These interpretations are, however, not similar across provinces. For instance, in some provinces it is required of committees to ensure sustainability and continued collaboration between all levels of the health system [37, 45].

#### Training of clinic committees

Certain provinces (37–40) have legislated some form of induction, training or capacity building, but it is not clear what these entail. The Ideal Clinic Manual, a reference guide for managers to enable them to determine the status of the Ideal Clinic dashboard elements in a facility, infers training to be the responsibility of the district [46].

In 2014, the Learning Network for Health and Human Rights developed training material called Health Committee Training: Participant Manuals. The Learning Network aims to use human rights to advance health issues through collective action and reflection, in order to identify best practices with regard to using human rights to advance health issues. The training manual, accompanied by a Facilitators Guide, is an on-going training and capacity building programme for clinic committees [47]. The programme has skilled facilitators. It provides a platform referred to as learning circles for committee members to share their experiences, consolidate new capacities and explore new topics [47]. The training is scheduled over three days and intended to be adaptable to different contexts, particularly with regards to legislation and needs of the participants [47]. This material has been in use since 2014, in the Western Cape and Eastern Cape provinces [42].

The ToF Learning Programme, the focus of this study and discussed elsewhere, had been commissioned by the NDoH and was meant to be a standardized training programme that could be adapted for different contexts [23, 48]. In referring to the ToF Learning Programme, the NDoH Annual Report for 2016/17 stated that ‘a Handbook with training material has been developed to institutionalise a uniform approach with regard to the establishment and sustainability of governance structures for PHC facilities’ [49]. The NDoH key informant, referred to in the interview results section, confirmed that the department’s overall plan is a national standardized dissemination of the training programme, by hosting one training session for representatives of the nine provinces and for these representatives to train facilitators within their respective provinces and districts, so that they in turn can train their clinic committee members (KI_5).

### Interview results

#### Participants’ perceptions of the training

Overall, the study participants (FGDs and DDoH KIIs) perceived the training as good. The managers regarded the training as challenging for the most part, of a very high standard and on par with tertiary level training. The facility supervisors found the training to be of a high, yet acceptable standard. The participants perceived the training tools and process to have facilitated the learning and transfer process. Some participants found the training schedule taxing due to the content and only one participant felt that more time should have been given to the training and that a follow-up session would have been helpful and allowed for further feedback. To this end, one of the NGO facilitators had indicated that the timeframe had already been increased from three days to five days, based on input and recommendations from a previous pilot.

Despite this, the participants perceived the training to have met its overall objective. They learnt how to train clinic committees. Unexpected to them the managers, in particular, felt that their public speaking, presentation and facilitation skills had been developed. Since the training, their own management roles were enhanced. The training exceeded their expectations and they were surprised at gaining knowledge themselves as training was meant to be of relevance to the clinic committees. Another unexpected outcome was that participants came face to face with the way they perceive themselves. They were challenged by their level of education and their abilities as managers. These perceptions differed between participants and some felt more strongly about it than others.

*“Well it was very, very good according to me because it was very informative. The material the scenarios everything was practical during training so it was easier for us to deliver to the clinic committee members and the nurse in our facilities.” FG2_P4.*

*“Whilst you are actually here to make sure that you impart knowledge to others, but you end up gaining yourself.” FG1_P3.*

#### Participants’ perceptions and experiences of clinic committees

The participants initially perceived clinic committee members to be less educated, old and slow to learn. Thus they were concerned that clinic committees would not be able to grasp the content and that the training would be beyond their grasp. In light of this, they were not sure whether and how the committee members would benefit from the training.

The ToF Learning Programme sparked some debate from its inception. Some of the study participants were concerned that the level of the training would be too difficult for the committee members to grasp. Others were, however, of the opinion that the intellect of committee members was being underestimated. One of the managers shared this concern prior to the training of the committee members, but after they had conducted the training he concluded that the ToF Learning Programme proved to be a source of empowerment for the committee members. These concerns were not unique to participants, as it was shared by the NDoH as well. Despite these concerns, the DDoH decided to use the training material as is, as they felt it was the duty of the DDoH to fully capacitate clinic committees and that training would signal to clinic committees that they are valued. Even though not part of the study’s initial inquiry, participants indicated that the facility managers, as members of the clinic committees, formed part of the clinic committee training. This was done to orientate facility managers on the roles and responsibilities of clinic committees.

*“I know that there was a bit of concern as well from the national department of health in terms of the content, but as a district we just felt let’s empower people to the best of our ability using the material that we have. Because in essence how then do you break it down further than it actually is? What do you say? I mean policy is policy…” KI_3.*

According to the focus group participants, their previously held perceptions of the clinic committees and the training had changed after training the clinic committee members. Two participants were particularly surprised that the committee members were able to understand and relate what they had learned to their daily activities.

*“When we looked [at] the material, we thought this is higher grade but when we actually start training, we could deduct that people understand. People know what is happening around their community…they could relate nicely.” FG1_P1.*

*“You might have elected them from ordinary community members by show of hand, but you must never underestimate their capabilities and their comprehension levels.” FG1_P3.*

Location and age profile of clinic committees seem to matter. Perspectives on clinic committee members were influenced by whether they were in a rural or urban setting. Participants were of the opinion that urban meant younger and more educated members, whilst rural meant older and less educated members. Those who conducted the training in urban areas experienced the training of clinic committees to be good. According to one participant, most clinic committee members were younger and could grasp quicker. There were, however, some committee members who struggled. One of the participants emphasized this:

*“We went to sub-districts. I also wanted to overemphasize on the issue of the knowledge and the skills of the clinic committees it’s not that low. You may think that these are just ordinary community members but when you get there you find very knowledgeable people. Though I admit that somewhere you find an elderly person one or two but the majority of them are young people and they can engage.” FG1_P3.*

Other reported impediments were language, education levels, and the time allocation of the training, which impacted on the delivery of the training. The participants who trained in the rural areas found that they had to translate the material from English into the local languages and found this particularly hard and time consuming.

*“The different language was exhausting because I had to change many times to all these languages.” FG2_P4.*

#### Capacity building of the trainers

Based on the FGD participants as well as one of the DDoH key informants’ own understandings of the training, it was felt that they themselves had been capacitated with the necessary knowledge and skills to train the clinic committees. The participants felt that their training capacity was strengthened, and at the same time, as a positive unintended consequence, their managerial capacity also got strengthened through the training programme, even though this was not the primary intention of the programme. In addition to this, the intended set of skills transfer, technical managerial skills such as conflict management and additional communication skills such as presenting, were enhanced. Even though one of the training facilitators was not entirely sure whether capacity strengthening took place during the training and if it did, to what degree, the participants from both FGDs and two key informants felt that capacity strengthening to deliver the ToF Learning Programme had taken place during the training.

*“As managers we also understood better on how we can deal with conflict that arise at a cold face and also issues that affect our staff.” FG1_P1.*

*“Really it polished our skills like presentation skills for some of us who did not know how to present.” FG1_P2.*

*“We gained knowledge and skills to be able to train the clinic committees.” FG2_P1.*

#### Empowerment of participants

Empowerment was one of the underlying aims of the ToF Facilitators guide. Ultimately the training is about empowering people. This means that stakeholders may need to make drastic changes should this tool no longer be capable of producing empowerment within people. Apart from being capacitated, a sense of being empowered resonated throughout, but this sense was more prominent in relation to the clinic committees. FG1 participants felt that through the knowledge they received during the training and the fact that they could pass this knowledge on to the clinic committees, that they had personally been empowered by the training. Following their (FG1) interaction with the clinic committees after their training, it had become clear to them that empowerment had indeed taken place. Another area in which participants felt empowered by the training was through knowing the roles of the governance structures at facility level. Prior to the training it was not clear to all the participants what the roles of the clinic committees were.

Whilst the study did not specifically verify the experience of clinic committees the trainers reflected from their perspectives that the clinic committees were empowered and these are some of the reasons they gave. According to the study participants the empowerment of clinic committees has meant different things. It has meant (a) members now had confidence to do what was required of them, (b) members’ functionality could lead to better health outcomes, (c) greater ownership of the health facility and communities, (d) better understanding of their roles and responsibilities as well as reporting lines, and (e) understanding management functions and administrative procedures. These came as a result of the participants’ interaction with the clinic committees since the training. One of the sub-district managers described the empowerment of clinic committees.

*“You will not feel that you’ve appointed clinic committees, until the training. After the training you realize they know their important roles in facilities. The training really changed how they see themselves and their authority. Now they started knowing that they’re heavy weights and they started punching on that level of weight that they carry. You can see the knowledge that they have and it changes your own perception.” FG1_P3.*

Whilst the study did not set out to determine the impact of the training, some participants volunteered their perspectives on possible outcomes; a change in certain indicators that they perceived were influenced by the training of the clinic committees. This could however not be corroborated as there were a number of factors that could have influenced these outcomes.

#### Ownership of health facilities

With the empowerment of clinic committees, the DDoH had somehow hoped that this would lead to a sense of ownership of health facilities. Based on discussions with participants, the act of empowering clinic committees had in fact led to a sense of ownership of health facilities and the communities amongst committee members. One of the participants relayed how, since training the clinic committees, the chairperson of a committee requested more involvement in decision-making processes at a particular facility.

*“They approach you to say please advise us on your recruitment process and some of them will even insist to say as a chairperson I want to sit in the interviews. I want to sit there and make sure that you get a good cadre there. I don’t want someone who’s going to wake up sick and it’s going to affect my population there.” FG1_P1.*

The notion of ownership of the facility has also been linked to participants fulfilling roles other than that of governance at facilities. One participant explained how community members take ownership of their facilities through doing what needs to be done at a particular time. Another participant likened ownership to the act of becoming ‘shock absorbers’ between the health professionals and the community.

*“If they have that passion and there’s that ownership, whatever challenges the facility is having they step in. And when it comes to the queue marshalling and so forth that’s part of the Ideal Clinic and with the shortage of staff they become very passionate and we do not discourage that. They are assisting in addressing the gaps that are there at that point in time” KI_3.*

#### Posters as valuable teaching aids for clinic committees

For the DDoH it was imperative that the facilitators had a solid understanding of the training material and content so that they could train the clinic committee members, irrespective of their level of education. The training material and the approach was designed to do just that, but the posters turned out to be most effective as a tool to capacitate the clinic committee members, as it broke each topic down to its simplest form. According to a key informant the clinic committee members found the posters most useful in educating their fellow committee members who were unable to attend the training sessions. This key informant also pointed out that in the absence of the manuals the clinic committee members were still well prepared to transfer their knowledge to their fellow committee members because they had the posters. The posters seemed to be enough.

*“Yes so it actually helped us a lot because you know even the type of material that was given to us you know big posters well laminated we could also give those to our clinic the governance structures, so that they can be able to display them in their rooms that they’re utilising and have their meetings, so that they can also be able to empower the other members that were not initially trained. But actually we expected everyone to be trained but you know some people are working they couldn’t all attend to our trainings but they could use those posters to be able to impart the knowledge after the training that they did in April and September the ones that we did. The follow-up training.” KI_4.*

*“You see if you’re teaching different people like them, maybe they didn’t have enough maybe writing material so that they can be able to explain and explain and explain, but they had the posters. The poster was assisting them. Yes, they had the means of doing it.” KI_4.*

### Printing of the training material

The DDoH indicated that it was dependent on external financial assistance, from NGOs, to print all the required training material to train the facilitators as well as the clinic committee members in the entire district. Without this assistance it is not entirely clear how materials, especially the posters which has now become integral to the district’s training, would be printed for future training sessions. The key informant indicated that the district has funding limitations.

*“They [NGOs] assisted us with the printing of all the training material. From the guideline, the facilitators guide to the participants guide to the posters.” KI_3.*

*“It’s just that maybe for us as a district to, how can I say, to develop a training manual again we will need a partner to assist us because it is very expensive. And if you see the quality of the participants’ manuals and the facilitators’ manuals, printed by [NGO], very beautiful you know. Pity I don’t have a copy now just to show you. So, for us it will just be, when we get the next term of clinic committees, how are we going to print all those posters because you know there’s funding [issues], but you have to remain positive. Maybe we’ll cross that bridge when we get there, but in terms of the quality of the training really I’m quite satisfied. KI_3”.*

### The role of stipends

Whilst not part of the study’s initial enquiry, stipends, an inductive theme, recurred throughout interactions with participants. The study did not set out to explore what impact stipends had on clinic committees’ motivation, however, from the participants’ perspectives, they felt that stipends would be a motivator. This will have to be verified in discussions with clinic committees in a separate study.

As part of the document review (in the previous section) the issue of stipends was explored. Three of the documents reviewed (Eastern Cape Provincial Health Act, No. 10 of 1999; Gauteng Department of Health Policy guidelines for the establishment and operation of Primary Health Care facility committees, Draft 1. 2009 and the KwaZulu-Natal Health Act No. 01 of 2009) [35, 39, 45] referred to some form of reimbursement for meetings attended, or hours spent on committee work, or travel reimbursement for meetings attended. Stipends are not standardized across the three provinces and the rest of the provinces are silent on the topic. The Colloquium concluded that support in the form of reimbursement is a government responsibility and should be considered [42]. This was also a sentiment from the study participants. Whilst the document review indicated a lack of attention to stipends in provincial legislation, the empirical data from this study confirmed the impact this has had on the functioning of clinic committees. Stipends were perceived as a motivating factor for joining and leaving clinic committees, despite the fact that stipends had not been offered (KI_3). The absence thereof has led to continuous discussions, re-nomination processes and re-training of members. According to the participants, the drive for stipends was more from younger members and those with political orientations. Older members, pensioners in particular, expressed less of a concern for stipends and the study participants believed this to be due to them receiving monthly pensions (KI_3, FG2_P2). The majority of clinic committee members are unemployed and see serving on committees as a means to career advancement. The effect of a stipend on participation in clinic committees can only be fully explored through an additional study where clinic committee members will directly participate. This study is only able to reflect the perception of facilitators drawn from their engagement with clinic committees during their training. Stipends are given to hospital boards and not clinic committees, despite both being governance structures. The absence of stipends, in particular, has served as a barrier to functionality, longevity and sustainability of active clinic committees (FG1_P3, FG2_P4 and KI_6).

From a government perspective, stipends have not been an easy topic as it has funding, governance and accountability implications (KI_6). The DDoH has expressed an awareness of the issues around stipends and is in the process of formalising it through having a unified approach (KI_5). To this end the NDoH has conducted a short study aimed at assessing the status of stipends in two provinces. On the other hand, the participants from the FGDs also questioned the volunteerism approach and felt that it leads to members with low educational levels and no prospects of career development (FG1_P3).

## Discussion and conclusions

Despite the deviation in the training process, the significance of the ToF Learning Programme is that it was still delivered as intended. The training fulfilled its overall purpose in that the facilitators’ capacity was strengthened and they were able to deliver the ToF Learning Programme to the clinic committees. The literature emphasises the importance and need for training clinic committees, yet without enough focus on the actual training of clinic committees. This study focussed largely on the training of facilitors or participants of the training who later trained clinic committees. Based on their perspectives, they reflected on the value of training for both the trainers and the eventual recipients, the clinic committees. Through the perspectives of the training programme participants, they posited that clinic committee members were receptive to and valued the training. They had examples of where the functioning at facility level seem to have been enhanced following the training. As shown in the results section, based on the views of the focus groups and key informant participants in the study, the ToF Learning Programme can thus be seen as beneficial and can be recommended for implementation. The results also suggest that the Programme is feasible, potentially sustainable and could be adapted to different contexts. Feasible, in that clinic committee members can be trained, including follow-up trainings, within a short period of time. The training of facilitators within and from the same districts speaks to its ability to be sustainable. Capacitating individuals from within the same districts and or areas have the ability to foster sustainability. Financial partnerships with NGOs have also been highlighted as measures towards sustainability as these would assist in printing of training materials, especially the posters. A number of factors contributed to the successful outcome of the training process.

### Trainer of trainer approach

Trainer of trainer courses are beneficial, highly effective, yield positive outcomes and influences attitudes and behaviours in that they tend to [1] be more cost effective than employing professional trainers [2] develops local capacity [3] maintain cultural significance and adaptation necessary for learning [12]. It is appropriate in settings where large numbers of people require training, bearing in mind the quality of the training material [12]. Trabeau et al., however, found no substantial difference between a trainer of trainer approach and training by an expert approach and suggests focus be directed to understanding the training program and the theory that guides it instead [15].

The sustainability of trainer of trainer courses is partly dependent on the measures of follow-up on trainings put in place as this determines future training and curriculum developments [12]. The ToF Learning Programme made provision for three follow-up sessions and the DDoH has continuous training done by facility managers. Sustainability is further impacted by programme design, the participant’s ability or readiness to learn, the trainers ability to train and accountability mechanisms through which all parties involved (trainers, trainees and management) adheres to [11]. It can also pose challenges if certain aspects such as resources, training environment, clear purpose of training and criteria for participants amongst others are not taken into account [12, 14, 15].

Factors enabling transfer are dependent on the process and characteristics of trainees; such as their ability to learn, the extent to which they are guided by their conscience, their choice in voluntary participation and the atmosphere in which they work [14]. Successful transfer is dependent on a combination of factors, namely, the trainers’ ability to conduct the training, and the trainers participation in the design, implementation and evaluation of the training process [13]. It is thus critical for trainers to have a concrete understanding of the underlying philosophy, the skillsets to transfer this understanding in creative ways, to have a clear understanding of the desired outcomes of the training and to conduct training in a manner that yields these outcomes [13, 15].

From the participants’ perspective and based on their interaction with governance structures the latter were capacitated, empowered and have shown the ability integrate this new knowledge into their day to day activities. With regards to sustainability and continuous capacitation this approach may need to be considered as an alternative to the policy intent. This confirms that training programmes with a systematic plan and direction can be sustainable and effective for clinic committees [22]. On-going sustainable training programmes can serve as a means of continuous capacitation [20].

### Adult education theory

The process of adults learning is often referred to and seen as being transformative and transformational [25, 50, 51]. Apart from acquiring various sets of knowledge and skills, it enables a deeper understanding of self and one’s being in the world [52]. This understanding is reached through the learning experience that encourages the engagement of one’s own views, experiences and understandings of the world [52]. The differing pre and post perceptions of the training that participants have of themselves and others are indicative of the power of adult education to be both transformative and transformational. At the same time, adult education remains complex, ever changing and requires attention to the context within which learning takes place [25].

### Training of managers

Studies have shown the value-add of capacitating health managers and how it can contribute to better health outcomes and organisational change [53, 54]. Through the ToF Learning Programme, manager’s technical managerial skills such as conflict management and additional communication skills such as presenting, were enhanced. The participants indicated that the training improved their knowledge about clinic committees and their understanding of the roles and functions of clinic committees. Their skills, facilitation and public speaking, improved and they are able to better perform their managerial tasks. An unintentional consequence of the ToF Learning Programme was that it strengthened the managerial capacity of the participants. This was not the primary intention of the programme. Further to this, capacity building efforts that have government support and positive management attitudes, can contribute to the success of these efforts [55]. Contextual factors [56, 57] including local as oppose to external trainers can impact training successes significantly.

### Policy

In South Africa, the establishment of clinic committees is a national requirement, as indicated by its national and provincial legislatures. These legislatures however do not prioritise capacitating members of these committees. Policies are essential, but often inadequate when it does not address all that is required for transformation [58]. Hence deliberate policy changes, including the development of skill sets, are required for the achievement and sustainability of health goals [59–61]. Lack of clear national level guidelines indicates a gap in policy development, interferes with and can lead to inconsistent implementation [62, 63]. Policies thus have to adequately reflect and prioritise desired health outcomes. These have to be in place for successful roll-out of national strategies.

### Stipend

Volunteerism, a vehicle through which PHC is delivered requires a change at health systems level that promotes community participation in health [64]. In the South African context, in particular, the concept of volunteering has evolved into a service associated with some form of remuneration [65]. Volunteerism was envisioned as a form of job creation and alleviating poverty [65]. The lack of stipends or other forms of reimbursement demotivates community participation in health and often leads to high attrition rates [66]. The role of stipends cannot be underestimated in volunteerism. It serves as a major motivator for involvement and the lack thereof impacts on the functioning, sustainability and stability of clinic committees. It also has negative implications for training. The policies reviewed showed an inconsistency with regards to stipends for clinic committee members.

### Perceptions of community members

The IEF was instrumental in drawing out intended and unintended consequences [32, 67]. The perceptions revealed towards the community members is one such unintended consequence as it points to prejudice of professionals which leads to them underestimating community members abilities and intellect. The IEF was useful in that it allowed participants the opportunity to reflect on their experiences as participants at first and later as facilitators. A limitation particularly pertaining to the IEF was that this was a retrospective study, which did not allow for direct observation and documentation of the processes as it unfolded. Reports and documents containing evaluation of the training were not obtainable, even though these were requested from relevant authorities.

## Limitations and opportunities of the study

The successful transfer of training could not be tested as the clinic committees did not form part of this study. This lends the opportunity to further enquiry that will include clinic committees. Perceptions of participants about the benefit of the training to clinic committee members will have to be corroborated with clinic committees at a later stage. The low participant response rate may have introduced selection bias. The reasons for the low response rate are unknown. However, the participants readily offered both positive and negative reflections on the training, thus the responses were not biased in one direction only. Document reviews have limitations. These include by nature of its design, limitations to required information, challenges with accessing documents and it may be biased in the way documents are selected [33]. As is the nature of qualitative research, the initial methods set out for this study did not unfold quite as expected. In qualitative research, a change of plans can always be anticipated and as a researcher one need to be flexible, bearing the original research design in mind [68].

## Recommendations

### Policy recommendations

This study set out to assess whether the training model presented in a South African district works and to make recommendations for future training. Based on the results of this study; the documentary review and the interviews, a few implications for policy emerged.

#### Legislative prioritisation

The current inconsistent legislation and application of training suggest that standardised legislative guidelines for the ToF Learning Programme are required. The development of a standardized national guideline for governance structures is critical. This will further communicate clinic committees as a national prerogative and give a unified direction with regards to clinic committees. It will also aid the process of monitoring and evaluation of the committees.

#### Standardisation of the training

Large scale uptake of the ToF Learning Programme will require a standardisation of the training programme, but this must be accompanied by flexibility so as to allow for differences in district contexts.

#### Training availability and accessibility

In order to enable the wide dissemination of the ToF Learning Programme the necessary processes and resources should be in place to make training available and accessible to all committee members across the nine provinces.

#### Stipend

Based on the concerns about stipends, a national position on stipends is required and must be fast-tracked considering the impact of lack of stipends on clinic committees’ functionality.

### Practice recommendations

Based on the results of the study and what this may imply for policy, the following recommendations on the length and format of training through the use of train-the-trainer approach are advised to aid future training.

#### Language

Training material should be translated into the different languages which represent the South African population to foster inclusiveness and encourage learning.

#### Greater inclusivity

Based on the results from the training conducted in this South African district it would be highly advisable to extend the ToF Learning Programme to all levels of the health system (national, provincial, district, sub-district and facility) and the clinic committees. This has the potential to bridge the gap currently existing between the department of health and the community and serve to familiarise all levels of the health system with the roles of clinic committees.

#### Training material

In order to make the training more effective and sustainable it would be advisable to print only the 16 Posters and the Pocket Handbook. These are to be made available at facilities for the facilitators and committee members for the purpose of training, reflection and use during follow-up sessions. This will allow for greater access to the material and for the DDoH to make this possible.

## Supplementary Information

**Additional file 1.** Study questionnaires (KII and FGD)

## Data Availability

The study data are available from the corresponding author on reasonable request.

## Abbreviations

COPASAH: Community Practitioners on Accountability and Social Action in Health
DDOH: District Department of Health
EQUINET: Regional Network for Equity in Health in East and Southern Africa
FGD: Focus Group Discussion
IEF: Illuminative Evaluative Framework
KII: Key Informant Interview
MEC: Member of Executive Council for Health
NDOH: National Department of Health
NGO: Non-Governmental Organisation
PHC: Primary Health Care
SA: South Africa
TOF: Trainer-of-Facilitator
