# Supplementary material for: An assessment of a ‘training-of-trainers programme for clinic committees’ in a South African district: a qualitative exploratory study
Source: BMC Health Serv Res. 2020 Nov 30;20:1101. doi: 10.1186/s12913-020-05921-z (PMC7702689; doi:10.1186/s12913-020-05921-z)
Supplement: Supplementary file 1 — Additional file 1. Study questionnaires (KII and FGD) [file 12913_2020_5921_MOESM1_ESM.docx]

# Key Informant Interview Questionnaire

| *For the interviewee to complete*  **Name of researcher: …………..……………………………………..**  **Name of supervisor: ………………………………………………….**  **Name of co-supervisor: ………………………………………………**  **Name of organisation: ………………………………………………..**  **Date of interview: …………………………………………………….**  **Venue of interview: …………………………………………………..** |
| --- |

**Interview process**

- Researcher formally introduces themself, reiterates study aims and goals, explain the interview process, and reason for participant’s selection.
- Take a moment to go through the consent form, again, clarify and answer any questions that may arise, ensure that participants understands what is being asked of them, that their participation is voluntarily and that they are free to stop participation at any time.
- Proceeds with interview and recording once participants consent to participate and consent forms are signed.

*[Press record on voice recorder]*

Thank you for agreeing to take part in this study *[ensure statement acknowledging consent is recorded].*

1. Can you please share a little bit about yourself; the position you hold and what you do in this position? *(Probes: What is your job title? How long have you been in this position? How does this relate to governance structures in the district?)*
2. Can you please tell me why the district DoH decided to conduct training for its master trainers? *(Probes: What were the external factors that influenced the decision? What were the internal factors that influenced the decision?)*
3. What macro and micro factors played a role in the commissioning of the training? *(Probes: Societal and political pressures and interests, Historical and socio-cultural, context, International context, Economic conditions and policy, The organisational climate and culture, Other policies and experiences, Organisational capacity)*
4. What is the Department of Health’s overall plan for training clinic committees in the district? *(Probes: Was this training a short-term plan? Are there long-term plans for training of clinic committees and master trainers? Are there any plans of scaling up to the rest of the Province?)*
5. Is training of clinic committees prioritised in the district’s budget? *(Probes: Is there an allocation of funds for training of clinic committees? Who is responsible for managing the funds? How is funding to clinic committees allocated?)*
6. Please tell me about your understanding of clinic committees. *(Probes: What are clinic committees; what function, if any do they fulfil? How does clinic committees relate to what you do? Why clinic committees? Who are its members?)*
7. How do clinic committees function in this district? *(Probes: What do they do? Is there a work plan that outlines day to day tasks and responsibilities? Who oversees them? Who do they report to/what are their reporting lines? How many clinic committees do you have in the district? Is every facility represented by a clinic committee?)*
8. Please tell me about your general understanding regarding the training of clinic committees. *(Probes: Why are clinic committees trained? Is training a priority? How often are clinic committees trained? What is the district’s plan for training of clinic committees?)*
9. Why did you decide to do a trainer-of-trainers’ model or type of training? *(Probes: What training model did you use before? Why did you not employ that model again for this training? Why did you decide on this specific training and material? Why did you choose HST as a service provider?)*
10. Has the training process been monitored since the initial training? Please explain. *(Probes: What has been monitored? Who has been doing the monitoring? What are the monitoring criteria?)*
11. How were candidates selected to attend the training? *(Probes: What criteria for selection were used? Who attended the training? Why were these initial attendees selected to attend?)*
12. How did you find the training itself? *(Probes: What was your overall impression with; trainers, material (content), timeframes?)*
13. What aspects of the training would you have done differently? Please explain. *(Probes: What information did you find extremely useful? What information did you find less useful? What are your thoughts on the timeframe of the training?)*
14. What has changed since the training? *(Probes: Has training been transferred to the clinic committees? Which clinic committees/sub-districts has been trained? How many clinic committees have been trained? Do you have any written or verbal feedback from the committees as to how they have experienced the training?)*
15. Do you have any suggestions as to how this training can be done differently or better in the future? *(Probes: Which aspects of the training would you leave out? Which aspects of the training did you find essential? What was your overall impression of the training? Would you recommend this training to other districts?)*

Do you have any questions that you would like to ask me?

Is there anything that I failed to mention and you feel is important to discuss regarding the training?

Thank You!

[Stop the recording]

# Focus Group Discussion Questionnaire

| *For the interviewee to complete*  **Name of researcher: …………..……………………………………..**  **Name of supervisor: ………………………………………………….**  **Name of co-supervisor: ………………………………………………**  **Name of organisation: ………………………………………………..**  **Date of interview: …………………………………………………….**  **Venue of interview: …………………………………………………..** |
| --- |

**Interview process**

- Researcher formally introduces themself, reiterates study aims and goals, explain the interview process, and reason for participant’s selection.
- Take a moment to go through the consent form again, clarify and answer any questions that may arise, ensure that participants understands what is being asked of them, that their participation is voluntarily and that they are free to stop participation at any time.
- Proceeds with interview and recording once participants consent to participate and consent forms are signed.

*[Press record on voice recorder]*

Thank you for agreeing to take part in this study *[ensure statement acknowledging consent is recorded].*

1. Can you please share a little bit about yourself; type of work you do, position you hold and how were you selected to attend the training? *(Probes: What is your job title? How long were you in this position at the time of the training? What was the selection process for attendees?)*
2. Please tell me about the training. *(Probes: Have you been part of this type of training before? How did you find the timeframe of the training? How did you find the way in which the training was conducted? How did you find the facilitators? What were the aims and objectives?)*
3. What are your thoughts on the name of the training programme? *(Probe: Was this easily understood?)*
4. Did the training change your knowledge about clinic committees in any way? If so, how? *(Probes: What was your understanding of clinic committees before the training? Is there anything you learned during the training that you did not know before?)*
5. What about the training did you find easy and what did you find difficult? *(Probes: Please explain easy. Please explain difficult.)*
6. How did you find the training material? *(Probes: Was it easy to understand? Was the language easy? Was the methodology easy? Did you find the material sufficient for what you needed to know in order to transfer the training? What was missing?)*
7. Since your training have you trained clinic committees? *(Probes: How many clinic committees have you trained? From which sub-district were these committees? Did you conduct separate trainings for separate sub-districts? Which methodology did you employ? Did you find it easy to transfer the training?)*
8. How many people attended the training? *(Probes: How many people were invited to the training? Of those invited how many attended? Were all attendees clinic committee members? What were the selection criteria for participants?)*
9. Which aspects of the training were easy to transfer? Why? *(Probes: Which aspects of the training were difficult to transfer? Why?)*
10. Do you have any written or oral feedback from clinic committees on their experience of the training? *(Probes: Which aspects of the training would you leave out? Which aspects of the training did you find essential? What was your overall impression of the training?)*
11. Do you have any suggestions about how this training can be done differently or better in the future? *(Probes: Which aspects of the training would you leave out? Which aspects of the training did you find essential? What was your overall impression of the training?)*

Do you have any questions that you would like to ask me?

Is there anything that I failed to mention and you feel is important to discuss regarding the training?

Thank You!

[Stop the recording]
